# Dental Prosthesis in Esophagus: A Right Cervicotomic Approach

**DOI:** 10.3390/life12081170

**Published:** 2022-07-31

**Authors:** Matteo Zanchetta, Elisa Monti, Lorenzo Latham, Jessica Costa, Alessandro Marzorati, Murad Odeh, Elisabetta Marta Colombo, Giuseppe Ietto, Davide Inversini, Domenico Iovino, Marco Paolo Maffioli, Luigi Fiorenzo Festi, Giulio Carcano

**Affiliations:** 1 Dipartimento di Medicina e Chirurgia, Università degli Studi dell’Insubria, 21100 Varese, Italy; elisa.monti.06@gmail.com (E.M.); j.costa200590@gmail.com (J.C.); giuseppe.ietto@asst-settelaghi.it (G.I.); davide.inversini@asst-settelaghi.it (D.I.); marcopaolo.maffioli@asst-settelaghi.it (M.P.M.); giulio.carcano@asst-settelaghi.it (G.C.); 2 Chirurgia Generale d’Urgenza e Trapianti, Ospedale di Circolo e Fondazione Macchi, 21100 Varese, Italy; lorenzo.latham@asst-settelaghi.it (L.L.); alessandro.marzorati@asst-settelaghi.it (A.M.); murad.odeh@asst-settelaghi.it (M.O.); elisabettamarta.colombo@asst-settelaghi.it (E.M.C.); domenico.iovino@asst-settelaghi.it (D.I.); luigi.festi@asst-settelaghi.it (L.F.F.); 3 Otorinolaringoiatria, Ospedale di Circolo e Fondazione Macchi, 21100 Varese, Italy

**Keywords:** foreign body, esophagus, right cervicotomy, cervicotomy, perforation, dental prosthesis

## Abstract

Foreign body ingestion in the upper digestive tract is a relatively common emergency. Less than 1% have to be treated surgically. We report the case of a 68-year-old man who ingested a dental prosthesis, probably during a seizure, and thus unknowingly, and presented two days later to the emergency department complaining of a mild dysphagia. A chest radiograph showed the presence of a removable dental prosthesis in the upper esophageal tract. The patient was brought to the operating room where a multidisciplinary equipe was assembled. Two attempts of retrieval with a flexible and a rigid endoscope failed because the removable dental prosthesis was stuck in the right pyriform sinus. Therefore, the surgeon performed an uncommon right cervicotomy and retrieved the foreign body through a right-side esophagotomy. The surgical approach depends on the nature and location of the foreign body. Urgent treatment is required whenever the patient develops dyspnea or dysphagia because of the high risk of inhalation and asphyxia. Removal of any esophageal foreign body has to be performed within 12–24 h. Repeated attempts to retrieve large dental prosthesis using an endoscope may result in esophageal perforation therefore when such risk of complication is too high, a surgical approach becomes inevitable. In our opinion, surgery remains the extrema ratio after a failed endoscopic retrieval attempt but can be lifesaving despite high risk of complications.

## 1. Introduction

Foreign body (FB) ingestion in the upper digestive tract is a common emergency that presents challenges to gastroenterologists, emergency department physicians, and surgeons. In daily practice, FB ingestion occurs usually in children, among which around 80% are between six months and six years of age [1]. Over the last twenty years, the incidence of FB ingestion in adults has risen from 3 to 5.3 per 100,000 persons in the United States of America [2]. According to the different mechanism, FB ingestion can be divided into accidental and intentional ingestion, the latter occurring mainly in prisoners and patients with mental or cognitive impairment [3]. In adults, the accidental ingestion of FB usually occurs while eating solid food [4].

The esophagus is the most common location in the gastrointestinal (GI) tract for FB obstructions and accounts for 57% to 75% of all impactions [5,6]. The cervical esophagus appears to be especially susceptible to FB impaction, possibly because the narrowest point of the GI tract is the cricopharyngeal sphincter, which is approximately 14 mm in diameter [7,8]. Esophageal impaction of FB requires prompt diagnosis and treatment because improper management may result in additional severe complications to patients. Of utmost importance is to determine the nature and precise location of the FB, the time passed since it was swallowed, and the likelihood of associated complications such as complete obstruction or perforation [7]. Patients who manifest clinical evidence of a rapidly worsening esophageal occlusion or who have ingested a sharp or pointed object require an emergent treatment due to the steeply increased risk of complications and exitus [8]. Although over 80% of FBs can pass through the GI tract spontaneously without intervention, 10–20% of FBs need to be removed by endoscopy and less than 1% have to be treated surgically [9,10]. The surgical approach depends on the nature and location of the FB [11].

We report the case of a 68-year-old man who accidentally and unknowingly ingested a dental prosthesis that remained stuck in his cervical esophagus and eventually required an unusual surgical operation to be retrieved, after multiple endoscopic attempts failed.

## 2. Case Presentation

A 68-year-old man presented to the emergency department (ED) of our Hospital (Ospedale di Circolo e Fondazione Macchi, Varese, Italy) complaining of a mild dysphagia for solid food that began after having a hot meal two days prior. He did not complain of any other symptom. Ideomotor slowdown, dyslipidemia and depressive syndrome were reported as the patient’s comorbidities.

The patient appeared calm and was eupneic, his vital parameters were normal, his Glasgow Coma Scale (GCS) 15. Upon physical examination there was no sign of any potential etiology, nor did he show any other clinical manifestation. Upon oral examination, some teeth were missing in the upper dental arch on both sides, and he denied any denture. Lab exams were within normal ranges. A SARS-CoV-2 nasopharyngeal swab test was negative.

**Figure 1:**
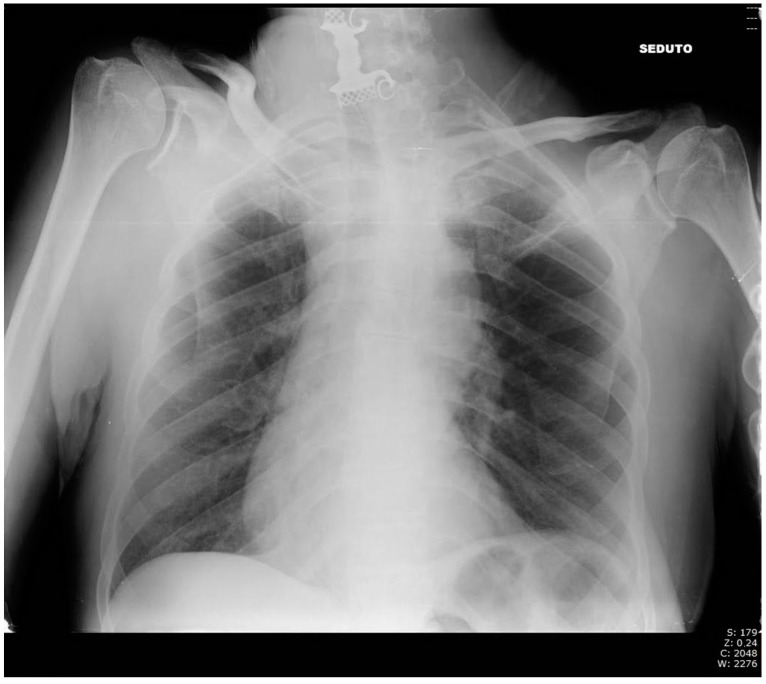
Chest radiography in the Emergency Room.

An otorhinolaryngologist was called for a consultation in order to obtain a specialist’s examination regarding the referred dysphagia. The specialist performed a laryngeal endoscopy that showed: edema of the right aryepiglottic fold; that the pyriform sinus was not visible due to salivary stagnation; that the right vocal cord was slightly edematous; and that the airway caliber was nonetheless deemed adequate. The otorhinolaryngologist, with laryngeal endoscopy being inconclusive, required the patient to be hospitalized to further study his case and properly manage it. In the meantime, the otorhinolaryngologist set up a therapy with corticosteroids and an antibiotic and gave the indication for a nil per os regimen. A chest radiograph was routinely done before hospitalization (Figure 1). The chest radiograph showed the presence of an FB projecting into the upper esophageal tract, slightly above the thoracic egress. The radiologist could not definitely identify the nature of the FB, although it was suggested it could be a dental prosthesis. When questioned about this, the patient once again denied any accidental ingestion nor dental prosthesis. The patient was therefore hospitalized in order to plan a proper therapeutic iter.

**Figure 2:**
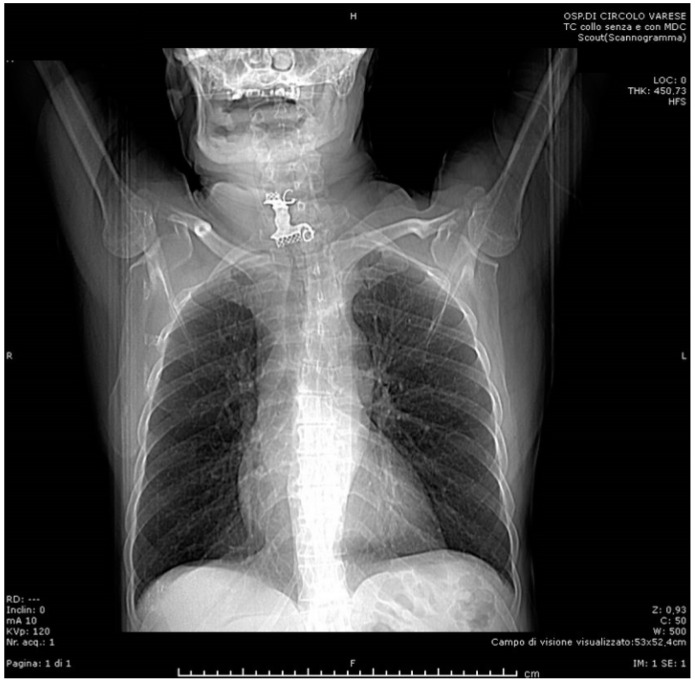
Preoperative CT scan (frontal).

**Figure 3:**
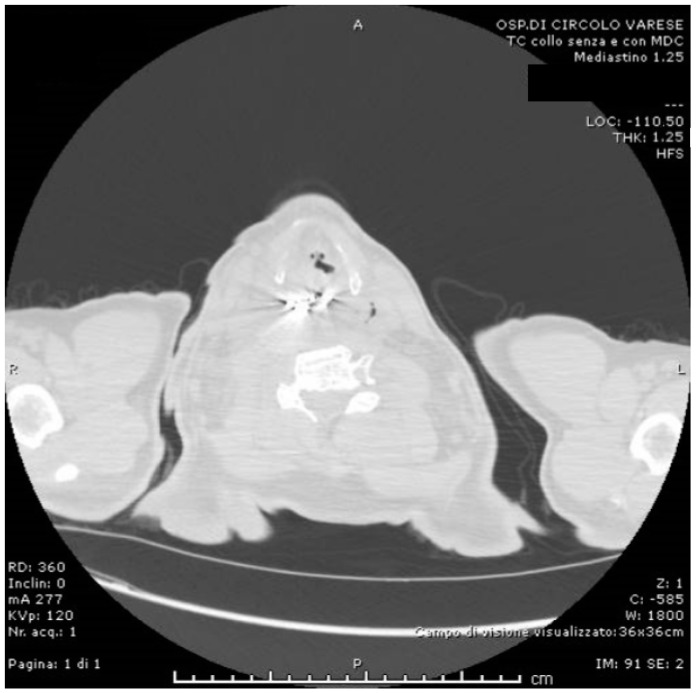
Preoperative CT scan (sagittal, lung and air view-window).

**Figure 4:**
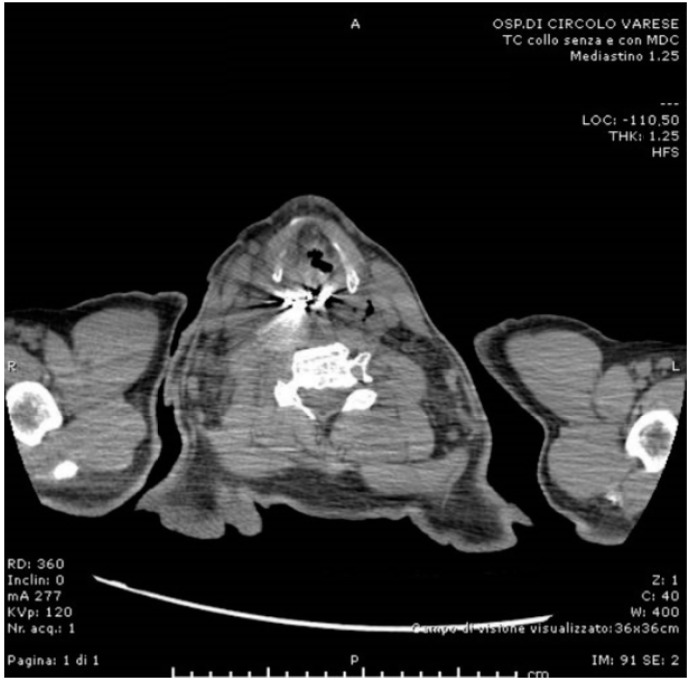
Preoperative CT scan (sagittal).

Soon after, the patient developed dyspnea and phonation difficulty due to a progressively enlarging swelling of the neck. An urgent neck and thorax CT scan was performed (Figure 2, Figure 3 and Figure 4), showing: a 5 cm radiopaque FB in the proximal esophagus at C4–C7 vertebral level; a few air bubbles in adjacency to the left cervical neurovascular bundle that suggested the presence of a limited esophageal perforation; swelling and thickening of prevertebral surrounding tissues; edema and thickening of the pyriform sinuses, especially on the right side; edema of the cervical esophagus with dishomogeneous contrast enhancement; numerous lymph nodes with reactive appearance in the laterocervical, periesophageal and mediastinal regions; and patency of the tracheobronchial lumina. The patient was therefore immediately brought to the operating room where a multidisciplinary equipe was assembled to firstly attempt a non-surgical approach, leaving the surgical retrieval of the FB as an extrema ratio.

The gastroenterologist using a flexible endoscope confirmed the FB to be a removable dental prosthesis with metal clasps, but did not succeed in its extraction. The otorhinolaryngologist, using a rigid Bouchayer endoscope, specified that the FB was inclined toward its right side and that it appeared to be stuck within the right pyriform sinus. Also, the second endoscopic attempt to retrieve the FB failed, because the FB was entangled in its distal part, therefore a forceful traction of it was deemed too dangerous. An operative approach became necessary.

**Figure 5:**
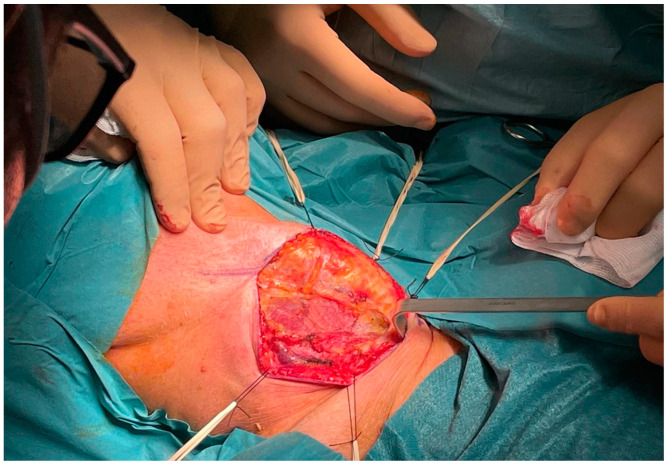
Intraoperative: cervical musculature.

**Figure 6:**
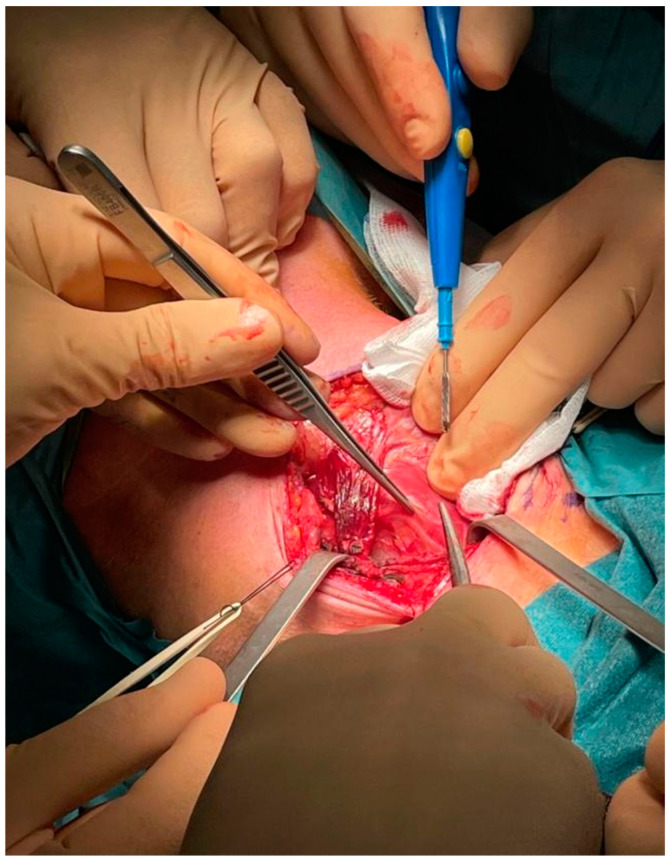
Intraoperative: esophageal wall.

**Figure 7:**
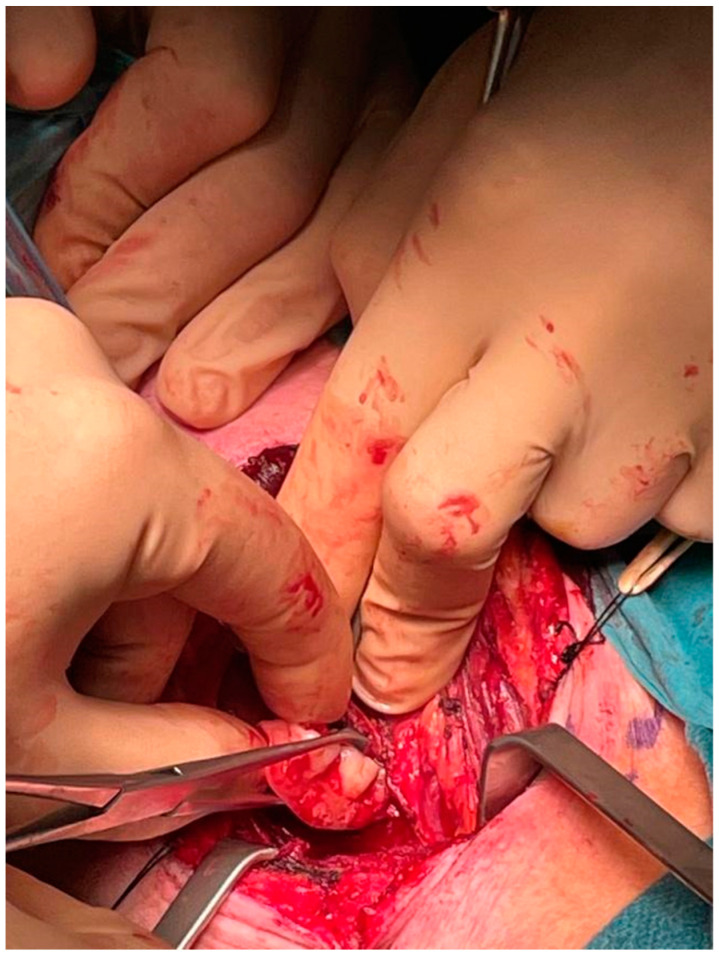
Intraoperative: extraction of the dental prosthesis.

**Figure 8:**
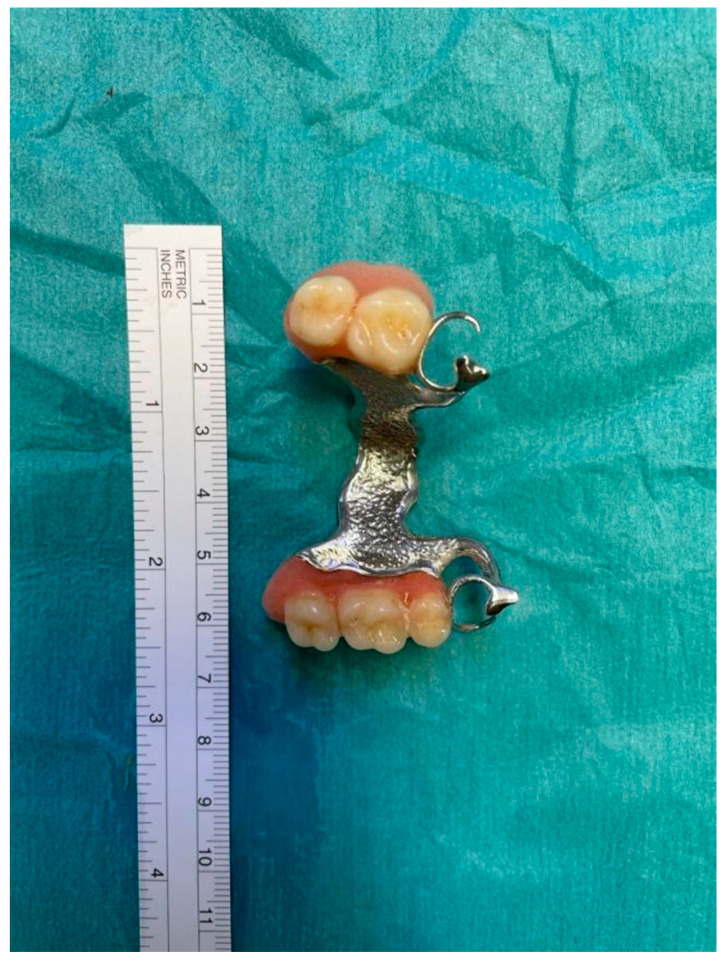
Removable dental prosthesis.

After a thorough inspection of the FB location, a right-side cervical approach was deemed more appropriate over the traditional left-side approach. The surgeon incised a 10-cm long right cervicotomy along the anterior margin of the right sternocleidomastoid muscle. The cervical structures were isolated (Figure 5) and moved aside until the esophageal wall was reached (Figure 6). Upon palpation, the surgeon clearly localized the FB within the cervical esophagus. The surgeon incised a 5 cm long esophagotomy and carefully retrieved the FB (Figure 7 and Figure 8) through it. A nasogastric tube was inserted under direct vision. After an accurate nonabsorbable two-layer continuous suture of the esophageal wall, a paraesophageal 19 Ch drain in aspiration was placed in situ. Superficial layers were subsequently sutured and reconstructed. An antibiotic therapy with piperacillin 4 g + tazobactam 0.5 g × 3/die was started.

From the surgical perspective, the postoperative course was regular. For the first week after surgery, the patient was fasted and supported with total parenteral nutrition (TPN). On postoperative day (POD) 7, methylene blue contrast was administered per os and no traces of any esophageal leakage nor stenosis were detected through the drain. Therefore, on POD 9, the patient was allowed to drink water and warm tea. There were no surgical complications in the following days, therefore on POD 14 the paraesophageal drain was removed.

**Figure 9:**
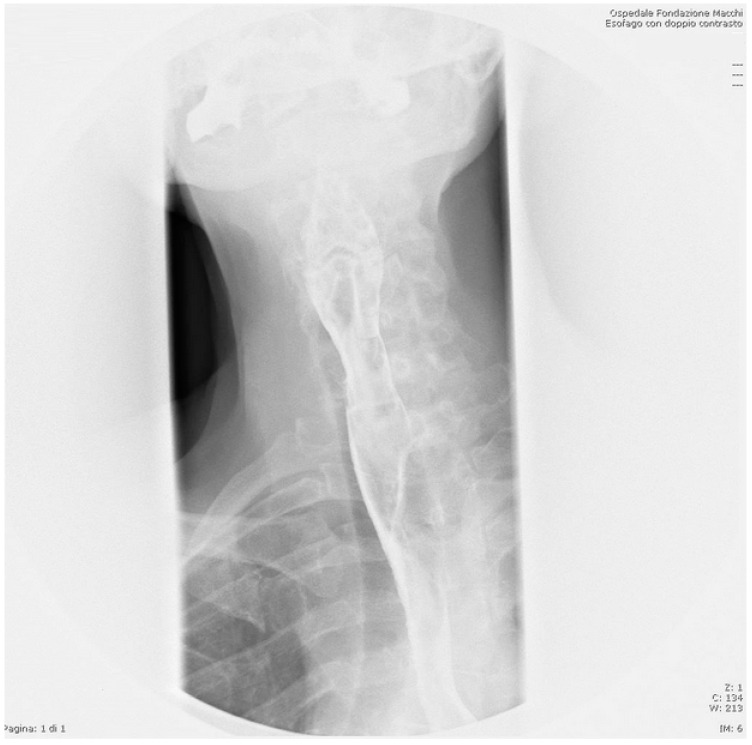
Esophageal medium contrast transit per os on POD 35.

During the first postoperative week the patient had several recurrent generalized tonic–clonic epileptic seizures with absence episodes. On the POD 17 these seizures worsened into a status epilepticus that required the transfer of the patient into an intensive care unit (ICU). Once the status epilepticus was resolved, he was transferred to a general medicine ward to continue the pharmacological management of the neurological condition. During the whole hospitalization, repeated neurological examinations and imaging studies ruled out any recent ischemic or hemorrhagic neurological event. Because of the recurring seizures and the risk they may have left sequelae, there was a major concern over the risk of suffocation as soon as solid food was permitted again. Therefore, a dynamic study of the oral and esophageal transit was ordered. On POD 35, an upper chest radiograph series enhanced with an oral transit of hydrosoluble iodine contrast medium showed: properly functioning deglutition mechanism, with complete and symmetric epiglottis movements; no extraluminal leakage of contrast medium from the proximal esophagus; and no stenosis of the esophageal lumen (Figure 9). The patient was allowed to eat solid food and no surgical complications developed in the following weeks.

## 3. Discussion

Any ingested FB is more likely to remain stuck within narrow areas along the esophagus [12]. The esophagus descends medially, along the column, with some physiological curvatures along its course. Because of the curvature at the cervical level, slightly toward the left, the cervical esophagus is traditionally approached through a left cervicotomy [13,14,15].

The ingestion of FB is a common yet challenging presentation for many physicians in daily practice. Despite mostly affecting the pediatric population [1,16,17], adults are not spared from this potentially severe occurrence. In adults, FBs are usually ingested accidentally together with food. Accidental ingestion or aspiration of FBs is commonly seen in the extremes of age (children and elderly) and in mentally and physically debilitated patients. The most common accidental FB ingestion in the elderly includes loosely fitted dentures, fish bones and meat boluses [18]. Contributory factors to such occurrence may indeed be psychiatric disorders, mental retardation, alcohol consumption, and an edentulous state [6]. However, in the setting of FB ingestion, impaction often occurs at the level of esophageal physiologic narrowings, but previous upper-GI surgery, congenital gut malformations, esophageal motility disorders or eosinophilic esophagitis may also represent significant risk factors [19].

In accordance with the literature, our patient had more than one risk factor related to FB ingestion: he suffered from depression, ideomotor slowdown, and had a dental prosthesis [7,20]. The neuropsychiatric condition of the patient may have slowed down the diagnostic and therapeutic iter, with the risk of potentially lethal complications, being the ingestion of FB a time-sensitive occurrence [6,21,22,23]. During the hospitalization he had several episodes of tonic–clonic seizures with absence that prolonged his hospital stay. We speculate that the ingestion of the dental prosthesis could have occurred during a seizure, leaving the patient unaware of the swallowing. Subsequently, the patient may have been unable to realize his denture was incomplete. It would not be the first case reported of lost dentures [24]. The diagnostic difficulty may be related to unclear history from patients with sensory deficit due to neurological conditions, cognitive deficit in older people, or lack of clinical signs [25]. It is worth noting that research has reported the misdiagnosis rate of denture ingestion to be as high as 47% [26].

Patients with an esophageal FB may be asymptomatic, symptomatic, or present complications. From a systematic review, 40 different studies have reported the location of the impacted FB in 7541 patients: there were 5044 FBs impacted in the cervical esophagus (66.9%), 1862 in the thoracic esophagus (24.7%) and 635 in the lower esophagus (8.4%). Retrosternal pain was the most commonly reported symptom (78%), followed by dysphagia (48%) and odynophagia (43.4%). Less frequently, respiratory symptoms (4%) were reported. A minority of patients (3.1%) were asymptomatic at hospital admission. An underlying esophageal disorder was diagnosed in 1872 of 7280 (25.7%) patients (26 studies). The most commonly associated esophageal disorders were stricture (33.9%), hiatus hernia (20.2%) and esophageal web or Schatzki ring (17.1%). Eosinophilic esophagitis was diagnosed at the time of presentation in 9.5% of patients [19].

Foreign bodies stuck in the upper esophagus carry a 25% higher risk of complication than other sites, further increased by the proximity of vital organs around it [27,28]. The most common clinical manifestations are dysphagia, odynophagia, hypersialorrhea, low cervical or chest strain, vomiting, and dyspnea when the trachea is compressed. Esophageal perforation is a severe complication that may either occur due to the shape of the FB or result from the handling and traction of the object during the removal. In our case, directly looking at the dental prosthesis with the endoscope, the equipe judged the risk of perforation to be excessively high for any further endoscopic attempt, thus opting for a surgical retrieval. The retrieval of any esophageal FB becomes increasingly difficult as time passes because of the local inflammation and reactive edema that develop around it. Other complications are retropharyngeal abscess, mediastinitis, and fistula [22], that may occur at later stages when the obstruction, erosion, or infection cause mucosal ischemia and necrosis resulting from prolonged impaction [10]. According to another study, the duration of the FB impacted in the esophagus is a significant factor associated with major complications; among the patients considered, 75% percent of those with major complications had their FBs impacted for longer than 24 h, while those with FBs impacted for more than 24 h were 14.1 times more likely than those with FBs impacted for less than 24 h to have a major complication [22]. Despite a two-day delay between the FB ingestion and the ED access, we managed to identify, properly evaluate and successfully extract it within 24 h from the moment of its detection [6,22,29]. Related risk factors for complications are time interval over 24 h between ingestion and ED access, positive radiographic findings, age over 50 years [23], involvement of the upper third of the esophagus, symptoms of complete digestive or respiratory obstruction, and high-risk objects due their shape, size, and composition [30]. Radiology, especially a timely CT scan [29], has a significant role in recognition of complications, possibly showing, for example, mediastinal, subdiaphragmatic, or subcutaneous air or pleural effusion [31], thickening of the soft cervical-mediastinal tissues, and presence of prevertebral emphysema [32], all suggesting perforation.

Therapeutic flexible endoscopy is recommended as the primary approach, with rigid endoscopy as a second line [33,34]. Urgent (<24 h) flexible endoscopy is recommended for esophageal FB that are not sharp pointed, neither batteries nor magnets, nor causing complete obstruction [33]. Rigid endoscopy through rigid endoscopes should be considered in case of FB located in the upper esophagus, and in case of FB ingestion with concomitant respiratory symptoms or suspicion of FB in the upper airways [33]. A comparative study between flexible and rigid endoscopy has shown that the former has better results in terms of postoperative comfort and dysphagia (15% vs. 48%; *p* < 0.0001) [35]. The results in terms of successful rate of FB retrieval are similar between flexible endoscopy and rigid endoscopy, though the former carries a higher rate of minor complications, while the latter has a higher rate of perforation (0.0% vs. 3.2% *p* < 0.002) [36,37]. A meta-analysis comparing flexible versus rigid endoscopy for retrieval of upper esophageal FB showed that both were effective and safe, with similar success and overall complication rates (*p <* 0.06) [38]. Repeated attempts to retrieve sharp FBs such as large dentures, especially those carrying metal clasps, using a flexible endoscope may result in esophageal perforation, considering the difficulty in aligning their irregular shape with the esophageal lumen. When such risk of esophageal perforation is deemed exceedingly high, as it occurred in our case, a surgical approach becomes necessary. The failure of endoscopic removal is mainly caused by the presence of abscess and/or perforation, prolonged time since ingestion, and the type and size of the FB, all increasing the likelihood of need for surgical treatment [10].

The surgical approach, whenever necessary, depends on the nature and location of the FB (e.g., left lateral cervicotomy along the sternocleidomastoid muscle, right thoracotomy in space IV, V, VI, left distal thoracotomy or laparotomy for impaction in distal esophagus [11]), and the comorbidities and general condition of the patient [33]. From a systematic review, a surgical approach through cervicotomy or thoracotomy was necessary for 325 patients (3.4%) because of perforation, esophageal fistula or unsuccessful endoscopic treatment [39]. According to the literature, potential indications for surgical treatment include irretrievable FB, failure of endoscopy, proximity to vital structures (aortic arch), esophageal perforation, and other complications such as increasing obstruction with dyspnea, and mediastinitis [10,20,33]. Usually, surgery for the cervical esophagus relies on a left-side approach due to anatomical reasons, mainly the physiological curvature of its structure within the neck tissues. However, from a surgical point of view, the right cervicotomy represents an alternative yet equally valid approach to the cervical esophagus and does not carry an intrinsically higher risk of postoperative complications. In this case we opted for an unusual right cervicotomy because of two different anatomical reasons. As evidenced by preoperative CT scan, the shape of the FB had moved the cervical esophagus to the right side of the neck. Furthermore, the metallic clasp of the FB was lodged within the right pyriform sinus.

In accordance with international guidelines, we performed an esophagotomy with FB extraction and primary closure [33]. We placed in situ a drain in aspiration, performed a contrast study before its removal, and administered a broad-spectrum antibiotic throughout the clinical course [40].

According to a study on the management of esophageal perforation, despite a successful operation, the mortality rate remains high after surgery (12%, against a nonetheless higher 18% mortality in cases managed nonoperatively) [21].

## 4. Conclusions

Any esophageal FB may cause potentially fatal injuries, therefore it becomes mandatory to retrieve it within 12–24 h. An endoscopic approach should be the first therapeutic choice, with surgery being an extrema ratio requiring a multidisciplinary equipe. According to our experience, the surgical approach should be tailored to the patient’s features, imaging techniques, and an endoscopic examination of the FB. Most importantly, a left surgical approach to the cervical esophagus should not be regarded as dogmatic, and a right-side approach, being viable and equally effective from a surgical point of view, could be appropriately chosen when clinically indicated.

## Data Availability

Patients’ data registry of Ospedale di Circolo e Fondazione Macchi (VA).
